# Deep Learning Assisted Imaging Methods to Facilitate Access to Ophthalmic Telepathology

**DOI:** 10.1016/j.xops.2023.100450

**Published:** 2023-12-12

**Authors:** Andrew W. Browne, Geunwoo Kim, Anderson N. Vu, Josiah K. To, Don S. Minckler, Maria Del Valle Estopinal, Narsing A. Rao, Christine A. Curcio, Pierre F. Baldi

**Affiliations:** 1 Department of Ophthalmology, Gavin Herbert Eye Institute, University of California, Irvine, California; 2 Center for Translational Vision Research, University of California, Irvine, California; 3 Department of Biomedical Engineering, University of California, Irvine, California; 4 School of Information and Computer Sciences, University of California, Irvine, California; 5 Department of Pathology, University of California, Irvine, California; 6 Department of Ophthalmology, University of Southern California, Los Angeles, California; 7 Department of Ophthalmology and Visual Sciences, University of Alabama at Birmingham Heersink School of Medicine, Birmingham, Alabama

**Keywords:** Deep learning, Telepathology, Diffusion model, Artificial intelligence

## Abstract

**Purpose:**

To investigate the use of super-resolution imaging techniques to enable telepathology using low-cost commercial cameras.

**Design:**

Experimental study.

**Participants:**

A total of 139 ophthalmic pathology slides obtained from the Ophthalmic Pathology service at the University of California, Irvine.

**Methods:**

Denoising Diffusion Probabilistic Model (DDPM) was developed to predict super-resolution pathology slide images from low-resolution inputs. The model was pretrained using 150 000 images randomly sampled from the ImageNet dataset. Patch aggregation was used to generate large images with DDPM. The performance of DDPM was evaluated against that of generative adversarial networks (GANs) and Robust UNet, which were also trained on the same dataset.

**Main Outcome Measures:**

The performance of models trained to generate super-resolution output images from low-resolution input images can be evaluated by using the mean squared error (MSE) and Structural Similarity Index Measure (SSIM), as well as subjective grades provided by expert pathologist graders.

**Results:**

In total, our study included 110 training images, 9 validation images, and 20 testing images. The objective performance scores were averaged over patches generated from 20 test images. The DDPM-based approach with pretraining produced the best results, with an MSE score of 1.35e-5 and an SSIM score of 0.8987. A qualitative analysis of super-resolution images was conducted by expert 3 pathologists and 1 expert ophthalmic microscopist, and the average accuracy of identifying the correct ground truth images ranged from 25% to 70% (with an average accuracy of 46.5%) for widefield images and 25% to 60% (with an average accuracy of 38.25%) for individual patches.

**Conclusions:**

The DDPM-based approach with pretraining is assessed to be effective at super-resolution prediction for ophthalmic pathology slides both in terms of objective and subjective measures. The proposed methodology is expected to decrease the reliance on costly slide scanners for acquiring high-quality pathology slide images, while also streamlining clinical workflow and expanding the scope of ophthalmic telepathology.

**Financial Disclosure(s):**

Proprietary or commercial disclosure may be found in the Footnotes and Disclosures at the end of this article.

Telepathology is the practice of pathology from a distance. Experts use advanced telemedicine technologies to remotely review pathology images acquired from anywhere in the world. The telepathologists then provide consultation to specialists where the images originate. While developed countries enjoy digital telepathology tools, analog telepathology imaging may predominate in developing countries. Technologies essential to digital telepathology include virtual microscopy, ultrahigh-resolution slide scanners, advanced telecommunications technology, and high-resolution digital displays. Automated digital slide scanners are used critically for virtual microscopy because they produce virtual slide systems using large digital image files of a complete glass slide. By storing large digital image files on a computer server, pathologists with an internet connection anywhere can virtually navigate slides at multiple magnifications with high resolution. A significant impediment to implementing virtual microscopy everywhere is the cost of automated digital slide scanners which may cost $80 to $350 000 (i.e., USD).

Smartphone technologies have enabled developing societies and groups to benefit from digital technologies more easily,^1^ and there is potential to expand telemedicine capabilities through this technology.^2^ In recent years, the proliferation of smartphones has led to a trend of digitizing physical documents by simply scanning them with smartphones. The once widely used flatbed scanners have experienced a decrease in demand as individuals resort to using their smartphones for this purpose. Despite these technological advancements, there remain obstacles to overcome in terms of image resolution. Considering this, the present research aims to examine the effectiveness of super-resolution imaging techniques in enhancing the resolution of low-cost commercial camera-generated pathology slide photographs.

Super-resolution refers to the process of improving the quality of low-resolution images by upscaling them to higher resolutions. However, due to the existence of multiple output images for a single input image, image super-resolution is a challenging inverse problem. Moreover, the conditional distribution of output images given the input image is often complex and not easily described by simple parametric distributions like multivariate Gaussian. Regression-based methods with convolutional neural networks, which are frequently used in image processing, have been proposed to improve super-resolution imaging.^3^ While feedforward convolutional neural networks may work well for low magnification ratios, they typically lack the level of detail required for high magnification ratios.^4^ Thus, the complexity and ambiguity of image super-resolution require more sophisticated and nuanced techniques beyond simple regression-based approaches. Deep generative models, on the other hand, can generate highly detailed images by learning complex empirical distributions.^5^ Various deep generative models, such as autoregressive models, variational autoencoders, normalizing flows, and generative adversarial networks (GANs) have obtained good results on super-resolution tasks.^6^, ^7^, ^8^, ^9^ While a few years ago GANs used to be considered state-of-the-art for many image-generation tasks, GANs are notoriously difficult to generalize, and they suffer from training instability.^10^ In this study, we investigated the use of the Denoising Diffusion Probabilistic Model (DDPM), which recently outperformed GANs in a variety of image generation tasks.^11^

To evaluate the effectiveness of our proposed methodology, we curated a dataset consisting of high and low-resolution images of ophthalmic pathology slides, as depicted in Figure 1A, B. Subsequently, we employed a DDPM model to learn from the dataset and predict super-resolution images from low-resolution inputs, illustrated in Figure 1C, D. The performance of the generated images was evaluated using objective metrics. We then compared the DDPM model performance with a regression-based model and a GAN-based model trained using the same data. Finally, we investigated whether 3 ophthalmic pathologists (N.R., M.E., D.M.) and 1 expert ophthalmic microscopist (C.C.) could distinguish the ground-truth high-resolution images from synthetic super-resolution images generated by our approach.

**Figure 1:**
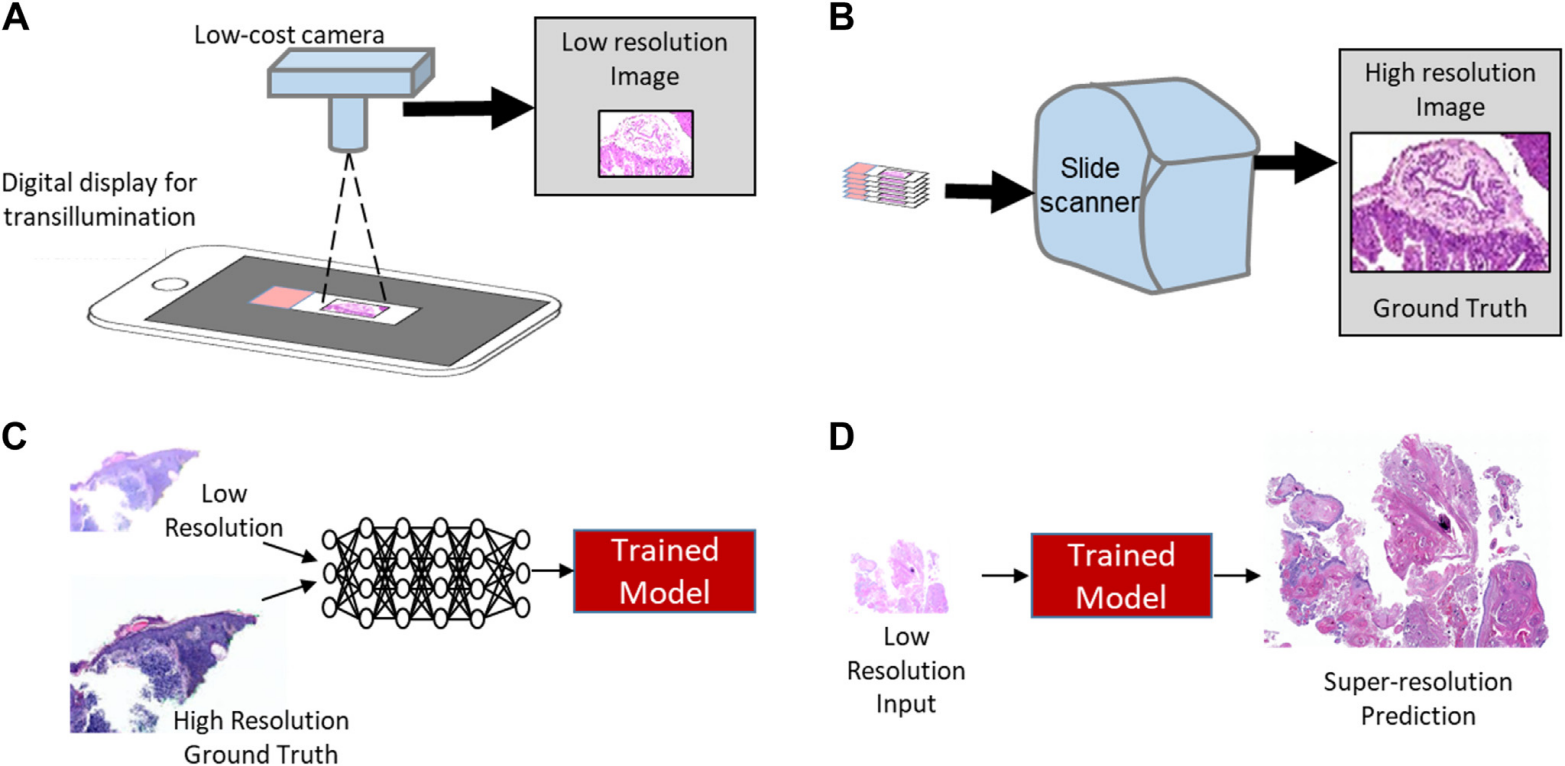
Description of overall workflow and project goals. **A,** Low-resolution images are acquired using a low-cost charge-coupled device camera to photograph slides placed on a digital display that provides white light transillumination. **B,** An automated slide scanner produces high-resolution images of the same slide. **C,** Paired low- and high-resolution images are used to train a model to predict high-resolution images from low-resolution input images. **D,** The model is tested and evaluated using objective metrics of image similarity (mean squared error, Structural Similarity Index Measure) and by evaluation by qualified observers.

## Methods

### Image Acquisition

**Figure 2:**
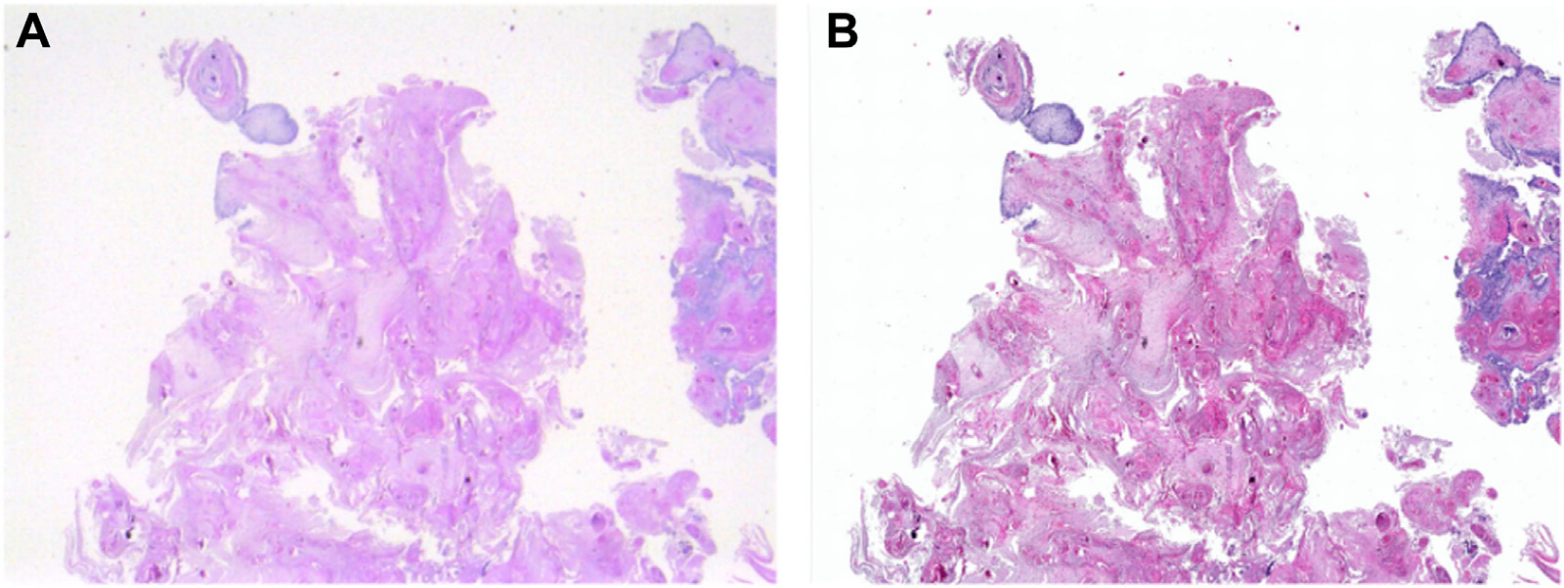
An example pair of images produced by a low-resolution Raspberry Pi Camera (**A**) and high-resolution Slide Scanner (**B**).

Ophthalmic pathology slides were obtained from the Ophthalmic Pathology service at the University of California, Irvine and used in accordance with approval from the institutional review board for retrospective research. Slides were scanned to achieve the highest resolution image on a Leica Biosystems Aperio Versa 200 (Leica Biosystems). Low-resolution images of slides were acquired using a 12 megapixel Raspberry Pi High-Quality Camera sensor and a telephoto lens. Slides were placed on a digital liquid-crystal display with a white screen background. Photographs of the slides were aligned and registered with high-resolution scanned images using i2k retina align software. One example pair of low-resolution images from the Raspberry Pi camera and the high-resolution image from the slide scanner is shown in Figure 2. The high-resolution images possess a pixel resolution of 3636 × 2727 while the low-resolution images possess 909 × 681 pixels. The dataset consists of a total of 139 image pairs, which were further categorized into 110 training images, 9 validation images, and 20 testing images. This study received institutional review board approval from the University of California, Irvine, and was conducted in accordance with the Declaration of Helsinki. Formal informed consent was waived by the institutional review board given the retrospective nature of the study.

### DDPM

To improve the image resolution, this study focused on using DDPM,^11^, ^12^, ^13^ which belongs to the category of deep generative models that generate data by means of iterative denoising. Specifically, the forward process of DDPM consists of generating random noise from data, whereas the reverse process was employed to generate data. This reversal was approximated through neural networks that maximize the data likelihood. Despite recent advances and the widespread availability of graphics processing units (GPUs), the memory requirements of DDPMs may exceed the GPU memory capacity if the input images are too large. Consequently, the use of DDPMs to recover super-resolution images from low-resolution images in our dataset was hindered by GPU memory limitations, forcing us to use multiple passes for a single image. Therefore, we partitioned the image into multiple nonoverlapping patches and ran the model separately on each patch. We then merged these patches to construct the complete image while addressing inconsistencies, especially at the boundaries, arising from independent model executions. To assess the quality of the super-resolution images produced by our model, we compared the patch-by-patch performance with 2 baseline models, namely Real-ESRGAN and Robust UNet.^14^^,^^15^ The models were trained using 8 NVIDIA RTX A5000 GPUs to accelerate the training process and facilitate batch processing of the diffusion model. To address the challenge of data scarcity, we pretrained the diffusion model with 150 000 randomly sampled images from the ImageNet dataset^16^ and subsequently fine-tuned it using our pathology slide image dataset.

For the pretraining phase, we employed the established super-resolution objective. Specifically, we sampled images from the ImageNet dataset and subsequently generated super-resolution images using the diffusion model. Given that our pretraining approach is conducted in an end-to-end fashion, the entire architecture of the network was trained. The ImageNet dataset is a widely used large-scale dataset in computer vision research. It contains millions of labeled images spanning over thousands of categories. By randomly sampling 150 000 images from the ImageNet dataset, we aimed to create a representative subset that captures diverse visual features and objects present in the larger dataset. The training was halted after 2 million iterations for our dataset and 6 million iterations for the ImageNet dataset.

### Data Processing

Initially, we performed bi-linear interpolation on low-resolution images to adjust their pixel resolution to match that of high-resolution images. The diffusion model was employed to recover resolution details from these interpolated images. As previously stated, the high-resolution images used in our investigation were of almost 4K resolution (3636 horizontal pixels and 2727 vertical pixels), posing a challenge for the training of the diffusion model using GPUs due to memory limitations associated with batch processing. Consequently, we randomly extracted 256 × 256 patches, obtaining a model output for each patch individually. We normalized the RGB values of the images to [−1, 1] to enhance training stability and the model’s ability to generalize.

### Patch Aggregation

It was not feasible to generate complete super-resolution images of entire images in a single iteration. Thus, we generated patches of images. Merging of the patches posed 2 significant challenges: (1) color disparity (e.g., variations in brightness and saturation), and (2) visible seams (i.e., discrepancies in recovered details). As the stochastic nature of the diffusion model precludes complete resolution of the challenges, postprocessing techniques must be applied to address these issues. To address the first issue, we generated multiple outputs for each pixel through the production of overlapping patches and averaged them to minimize variance. However, this method comes with an extended inference time, and thus, we limit the degree of overlap to 3, i.e., 3 outputs generated for the same pixel. In addition, we subsequently apply a Gaussian blur to the resulting image to smooth the hard edges between patches. To avoid a reduction in overall image resolution, the standard deviation of the blur was fixed at 0.5. Furthermore, we cut the patches into circular shapes to make the edges even more invisible.

### Evaluating Model Performance

The evaluation of a trained model's ability to predict super-resolution output using low-resolution input images was conducted using a combination of objective image metrics and subjective expert pathologist graders. The model's performance was objectively compared on individual patches, utilizing commonly employed metrics for image reconstruction, such as mean squared error (MSE) and Structural Similarity Index Measure (SSIM).^17^ To further evaluate the model's performance, 3 expert ophthalmic pathologists and 1 experienced microscopist were involved in the assessment. They examined 20 pairs of high-resolution images alongside their corresponding super-resolution counterparts. The experts were given the task of identifying which of the paired images was the ground truth high-resolution image. This assessment was conducted for both 20 full-frame image pairs and 20 individual patch pairs. Throughout the review process, the experts were kept unaware of the image sources to ensure unbiased evaluation.

## Results

First, we present a performance comparison between our DDPM-based approaches and 2 baseline models. Table 1 summarizes the objective performance scores of these models, which were averaged over the patches generated from 20 test images. The results indicate that the DDPM-based approaches produced lower MSE and higher SSIM scores than both Robust UNet and Real-ESRGAN. Additionally, the DDPM-based approach with pretraining showed further improvement in both MSE and SSIM metrics. A high SSIM score of 0.89 was attained by our DDPM-based approaches with pretraining, indicating a high degree of similarity between the compared images. Such a score implies that the images are almost identical, with only negligible differences that are hardly discernible to the human eye.

**Table 1:** Average MSE and SSIM of 4 Trained Models on Patches Generated From 20 Test Images

Model Name	MSE	SSIM
RUNet	0.076	0.5484
Real-ESRGAN	0.054	0.5860
DDPM	8.78e-5	0.8766
DDPM with pretraining	**1.35e-5**	**0.8987**

DDPM = Denoising Diffusion Probabilistic Model; RUNet = Robust UNet; MSE = mean squared error; SSIM = Structural Similarity Index Measure.

Bold values are from our proposed method and they achieved SoTA.

**Figure 3:**
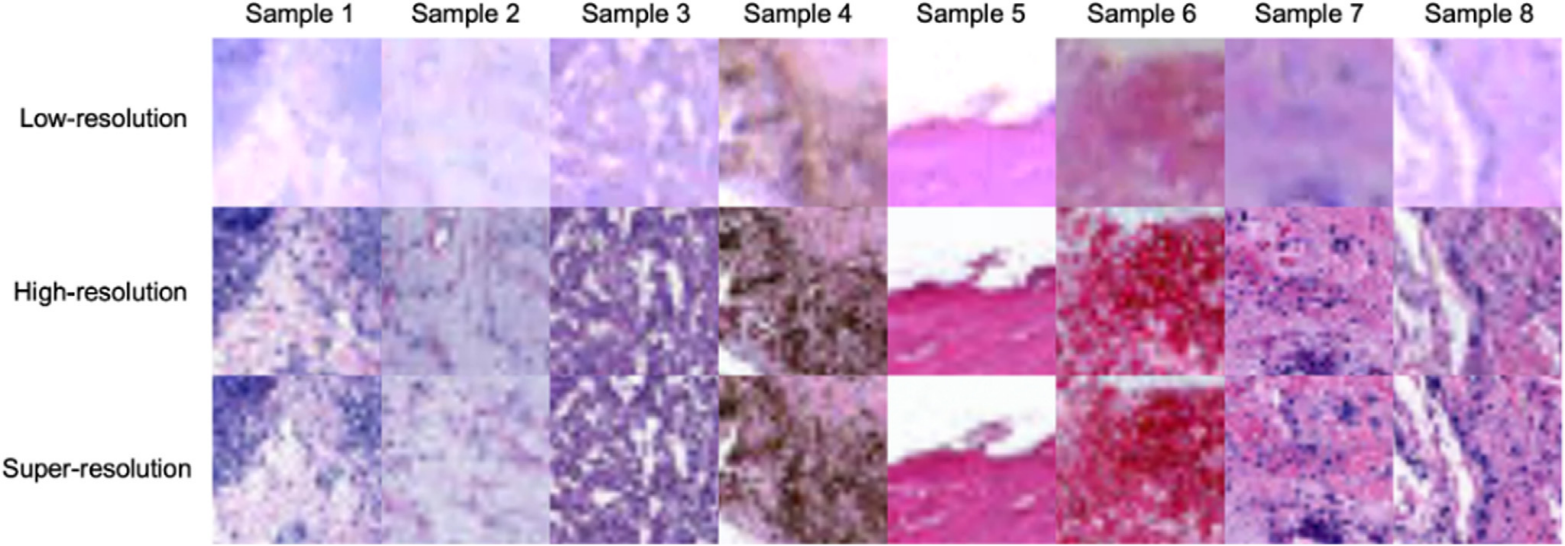
Sample patches of low-, high- and super-resolution images. Each column displays identical patches sourced from images of varying resolutions. Specifically, the first row pertains to low-resolution patches, the second row to high-resolution patches, and the final row to super-resolution patches generated through our proposed approach.

Figure 3 displays 8 examples of predicted super-resolution patches from the best-performing approach (i.e., DDPM with pretraining) along with their corresponding low- and high-resolution counterparts. The performance of DDPM was sometimes inconsistent in terms of recovering image brightness, as observed in Sample 2. However, in most cases, DDPM demonstrated success in restoring resolution details.

**Figure 4:**
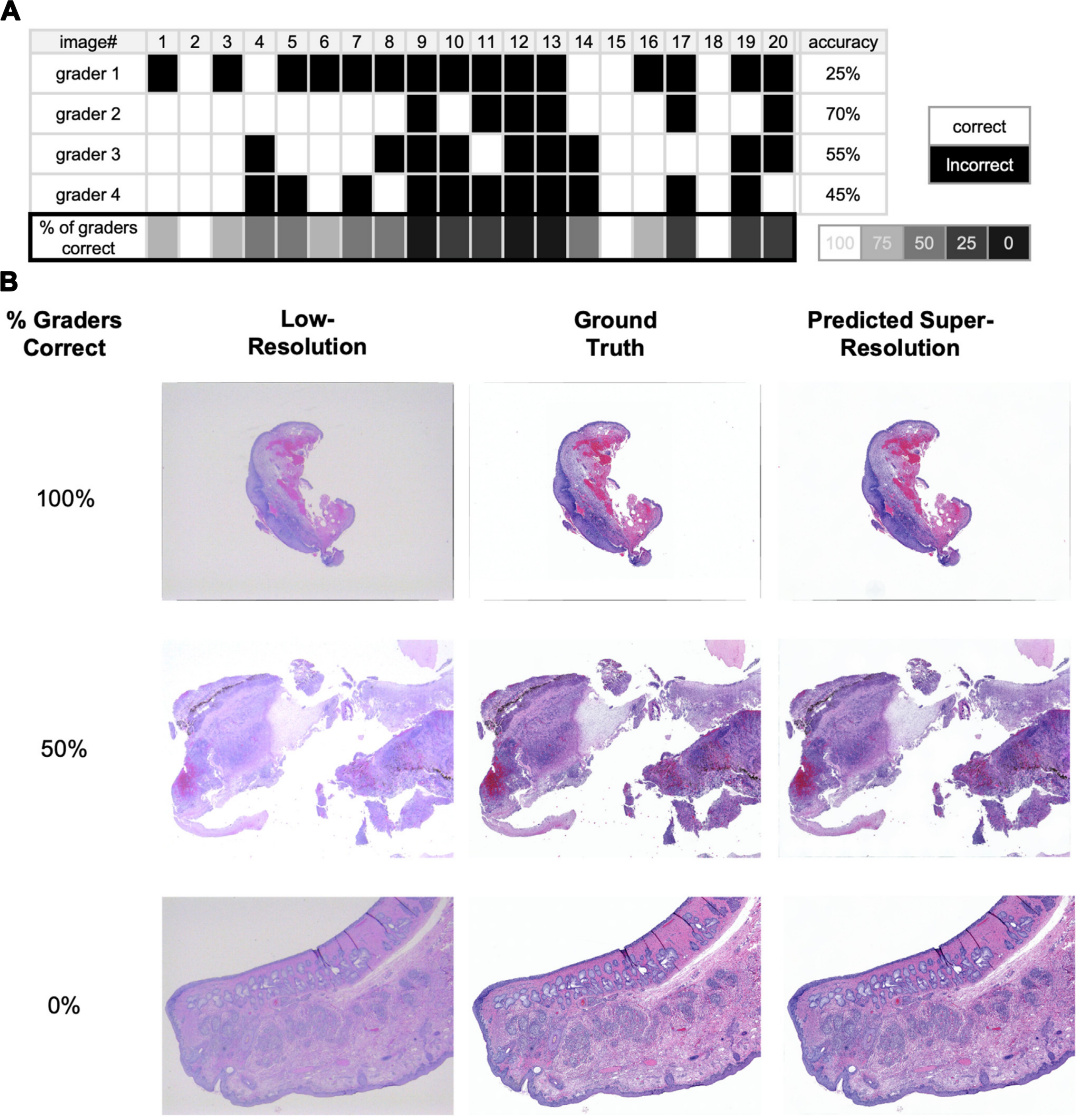
Results of test on 20 wide field of view microscopy images. Section (**A**) presents binary matrices illustrating the results obtained by graders for each of the 20 test images. The matrices show correct (white) and incorrect (black) answers. Panels in (**B**) of Figures 4 and 5 present visual representations of example images for which either most graders were accurate or most graders were inaccurate. It consists of 3 vertical columns of panels, with the first and second columns depicting low- and high-resolution images, respectively. The third column displays super-resolution images, which were generated by our model.

**Figure 5:**
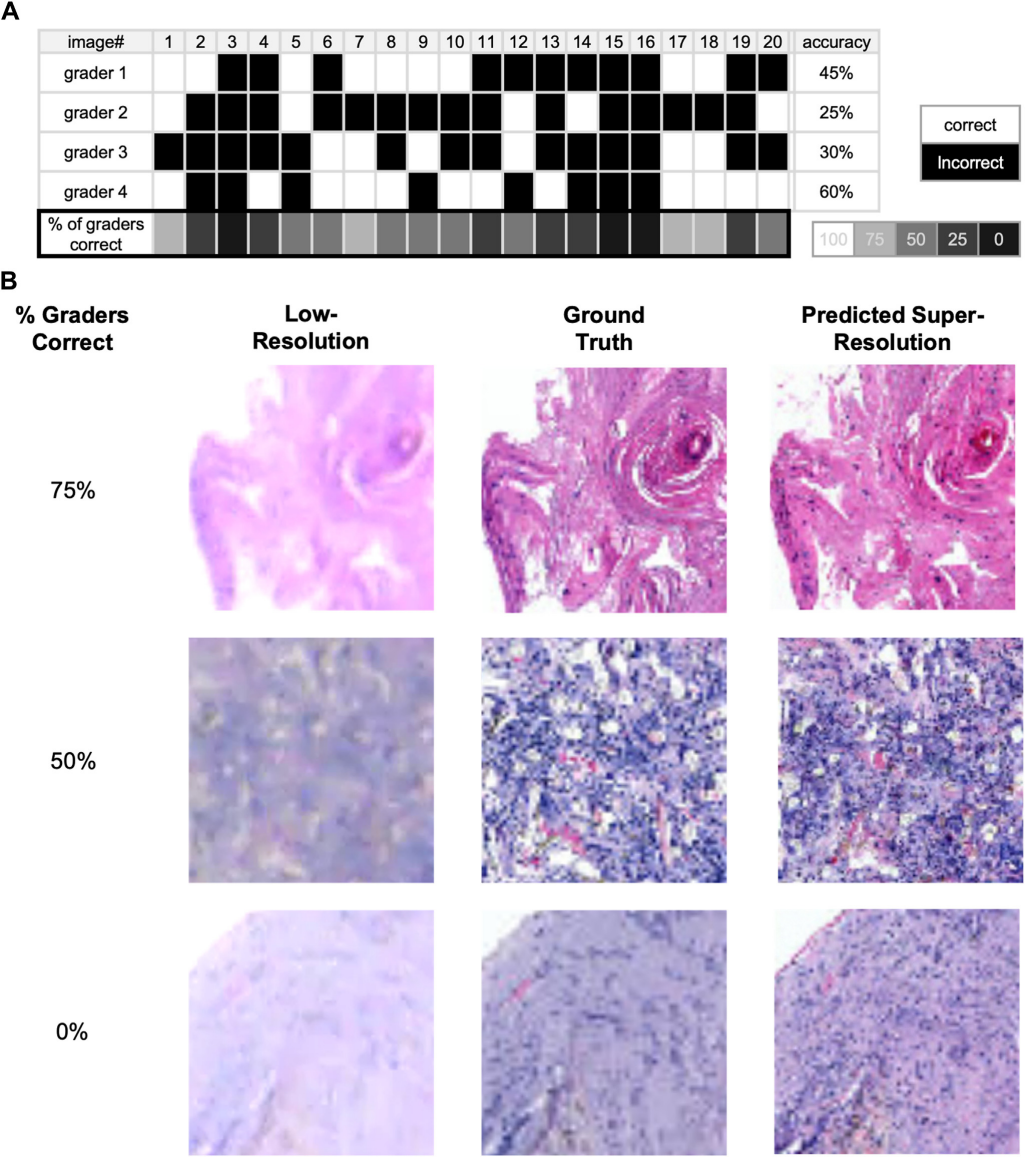
Results of test on 20 individual patches. Section (**A**) presents binary matrices illustrating the results obtained by graders for each patch sampled from the 20 test images. The matrices show correct (white) and incorrect (black) answers. Panels in (**B**) of Figures 4 and 5 present visual representations of example patches for which either most graders were accurate or most graders were inaccurate. It consists of 3 vertical columns of panels, with the first and second columns depicting low- and high-resolution patches, respectively. The third column displays super-resolution patches, which were generated by our model.

To further investigate the performance of our model, a qualitative analysis of super-resolution images was conducted to determine the extent to which ground truth images could be distinguished from predicted images by expert graders. The results of this analysis are presented in Figures 4 and 5, which depict the accuracy rate of experts tasked with distinguishing groundtruth images from super-resolution predictions. Specifically, Figure 4 pertains to wide field of view microscopy images, with the super-resolution image being composed of multiple stitched patches, while Figure 5 pertains to individual patches viewed at higher magnification. In both figures, section (A) presents the graders' results in binary matrices, with correct (white) and incorrect (black) answers for each of the 20 sample images shown. The average accuracy of identifying the correct groundtruth images ranged from 25% to 70% (with an average accuracy of 46.5%) for widefield images, and 25% to 60% (with an average accuracy of 38.25%) for individual patches. In addition, we presented the precision, recall, specificity, and F1 score for each grader in both qualitative assessments, as detailed in Tables 2 and 3. Panels in (B) of Figures 4 and 5 depict example images where most graders were accurate and none of the graders were accurate, respectively. These panels consist of 3 columns. The first column depicts images of low-resolution, while the second column displays high-resolution counterparts. The third column presents super-resolution images, which are the output of our model.

**Table 2:** Performance Metrics of 4 Graders in the Qualitative Evaluation on Widefield Images

Grader	Precision	Recall	F1 Score	Specificity
Grader 1	0.25	0.25	0.4	0.25
Grader 2	0.7	0.7	0.82	0.7
Grader 3	0.55	0.55	0.71	0.55
Grader 4	0.45	0.45	0.62	0.45

The table displays the precision, recall, F1 score, and specificity for each grader, providing insights into their respective accuracies and potential biases.

**Table 3:** Performance Metrics of 4 Graders in the Qualitative Evaluation on Patches

Grader	Precision	Recall	F1 Score	Specificity
Grader 1	0.45	0.45	0.6216	0.45
Grader 2	0.25	0.25	0.4	0.25
Grader 3	0.3	0.3	0.4615	0.3
Grader 4	0.6	0.6	0.75	0.6

The table displays the precision, recall, F1 score, and specificity for each grader, providing insights into their respective accuracies and potential biases.

## Discussion

We evaluated a workflow using a widely available camera to acquire photographs of ophthalmic pathology slides paired with high-resolution scanned images of the slides. We sought to evaluate DDPM as an alternative to Real-ESRGAN and Robust UNet to predict high-resolution images using low-resolution input photographs of slides. Our results demonstrated a substantial improvement in both MSE and SSIM scores with the use of DDPM rather than baseline models. This improvement can be attributed to the inherent ability of DDPM to capture and approximate complex conditional distributions between low-resolution and high-resolution images. Moreover, our analysis indicates that the DDPM model can recover not only the resolution from low-resolution images but also camera-specific features such as hue, saturation, and brightness, which are significant contributors to the overall appealing quality of the super-resolution images. Specifically, we observed that enhancing the contrast and brightness of the generated images aligns them more closely with the high-resolution ground truth images, resulting in better quantitative metrics scores. The findings of our study thus suggest that DDPM is a promising technique for generating high-quality super-resolution pathology slide photographs with improved visual and quantitative performance.

The efficacy of pretraining with ImageNet dataset on the performance of DDPM is noteworthy. While the diffusion model exhibited remarkable performance with limited data, our results demonstrated that pretraining can further enhance model performance. The use of small datasets to train models is a common challenge in biomedical imaging, which underscores the significance of exploring techniques that can boost model performance with limited data. Despite the modest gains in objective performance metrics observed in this study, our results highlight the practical utility of pretraining in addressing the limitations posed by small training datasets. This study provides compelling evidence supporting the adoption of pretraining as an effective strategy to improve the performance of DDPM in scenarios where data is scarce. It is our contention that this outcome will further augment the feasibility of our methodology and render it more practical for clinical settings.

After training our models, we then sought to evaluate human expert observation of predicted images as they compare with ground truth high-resolution scanned images. A human review of widefield images revealed that the observers could distinguish the ground truth image with an average accuracy of only 46.5%. While this accuracy rate indicates that super-resolution predicted images are subjectively similar in appearance to ground truth images, there is still ample opportunity to improve the patch aggregation processes to improve widefield image composition. Specifically, our analysis revealed that poor-quality patches located at the edges of images were the primary contributors to lower accuracy rates, seen as subtle artifacts in the super-resolution image of the first sample in Figure 4B. Additionally, despite applying Gaussian blur and averaging over patches, residual resolution disparities persisted. To address these limitations, we anticipate that future research efforts could explore (1) increasing the number of outputs per pixel and (2) implementing more refined patch aggregation methods. By elevating the number of outputs per pixel, we can achieve better consistency between patches by reducing the stochastic of model outputs. In addition, implementing more refined patch aggregation methods could help to address the disparities arising from factors beyond resolution. Overall, these potential solutions hold promise for more realistic super-resolution outputs of widefield images.

The presented pilot study also reveals imaging and model limitations. Image acquisition using a low-cost camera can suffer from image parallax. To minimize parallax, we used a telephoto lens to zoom on the slides when they were imaged. Slide scanners do not suffer from image parallax because the slide is located between and in close contact with the camera sensor and the light source. Second, registration of high- and low-resolution images using software may suffer from microscopic misalignments that negatively affect the model performance. While image parallax and registration may not be significant at the ultrastructural pathology specimen level, it is likely to negatively affect small cellular features. This may be overcome with refined hardware for low-resolution imaging or by using simple flatbed scanners that more closely approximate high-resolution automated scanners. The second group of limitations rests with model training, performance, and image reconstruction. First, a nonnegligible amount of computing resources is needed to run the inference of the diffusion model at a reasonable speed. In our experiments, we used 8 GPUs, but this might not be feasible in real use cases. In addition, the stitching method for generating the entire super-resolution images inherently suffers from visible seams and color discrepancy. One possible future approach is to explore recent improvements in diffusion models that accelerate the sampling process and reduce the stochasticity in it. However, their applicability to a wide range of data has not been proven, so more investigation is needed. Alternatively, further model exploration can be conducted to determine the model that does not involve probability sampling but can match the performance of the diffusion model. The proposed system for telepathology will need to be further enhanced with larger training datasets and validated in actual workflows for diagnostic accuracy by pathologists evaluating images produced by conventional and super-resolution imaging approaches.
